# Improved low-rank matrix recovery method for predicting miRNA-disease association

**DOI:** 10.1038/s41598-017-06201-3

**Published:** 2017-07-20

**Authors:** Li Peng, Manman Peng, Bo Liao, Guohua Huang, Wei Liang, Keqin Li

**Affiliations:** 1 College of Information Science and Engineering, Hunan University, Changsha, Hunan 410082 China; 2 College of Computer Science and Engineering, Hunan University of Science and Technology, Xiangtan, Hunan 411201 China; 3 College of Information Engineering, Shaoyang University, Shaoyang, Hunan 422000 China; 4 Department of Computer Science, State University of New York, New Paltz, New York 12561 USA

## Abstract

MicroRNAs (miRNAs) performs crucial roles in various human diseases, but miRNA-related pathogenic mechanisms remain incompletely understood. Revealing the potential relationship between miRNAs and diseases is a critical problem in biomedical research. Considering limitation of existing computational approaches, we develop improved low-rank matrix recovery (ILRMR) for miRNA-disease association prediction. ILRMR is a global method that can simultaneously prioritize potential association for all diseases and does not require negative samples. ILRMR can also identify promising miRNAs for investigating diseases without any known related miRNA. By integrating miRNA-miRNA similarity information, disease-disease similarity information, and miRNA family information to matrix recovery, ILRMR performs better than other methods in cross validation and case studies.

## Introduction

MicroRNAs (miRNAs) comprise a set of 22-nucleotide long, noncoding RNAs, which are widespread in fauna and flora^1^. miRNAs act as crucial regulatory factors of gene expressions that result in post-transcriptional repression or degradation by complementarily binding to specific 3′ untranslated regions of their mRNA^2^. miRNAs participate in various important biological progresses, such as cell survival, apoptosis, differentiation, tumor growth, and metastasis^3^. Therefore, abnormal regulation of miRNA may lead to development and progression of various diseases, including cancer^4, 5^.

Substantial evidence from over 24,000 peer-reviewed reports revealed that miRNA performs a crucial role in cancer, as mentioned in the paper written by Ganju *et al*.^6^, in which they summarized miRNA nanotherapeutics for cancer. Numerous miRNA-disease interactions were revealed over the years. Li *et al*.^7^ and Jiang *et al*.^8^ collected data from experiments that invalided miRNA-disease interactions and constructed two online databases, namely, human miRNA-disease database (HMDD) and miR2Disease, respectively. Yang *et al*.^9^ set up another publicly database named Differentially Expressed MiRNAs in Human Cancers (dbDEMC).

However, known associations between miRNAs and disease are still currently limited. Revelation of potential relationship between diseases and miRNAs is a critical problem not only in uncovering molecular mechanisms of various diseases but also in providing underlying biomarkers for disease diagnosis, treatment, and drug design. Biological experimental methods for finding disease-related miRNAs are expensive and time consuming. With accumulation of available studies and emergence of large amounts of biological data about miRNA, powerful computational approach can be used to mine underlying miRNA-disease associations from these data^10^. Computational approaches sort the most plausible miRNA candidates for further analysis, hence markedly improving efficiency of experiments.

In recent years, numerous approaches were presented to predict miRNA-disease associations from machine-learning-based and network-similarity-based perspective. Jiang *et al*.^11^ presented a computational model based on hypergeometric distribution to prioritize microRNAome candidates for predictive diseases to verify potential disease-associated miRNAs. Shi *et al*.^12^ utilized functional relationships between miRNA targets and disease genes to mine miRNA-disease associations. Mork *et al*.^13^ developed a miRPD method by combining known miRNA-protein associations to identify diseases-related miRNAs and underlying related proteins. However, the above methods strongly depended on miRNA-target associations, and their prediction performances are affected by high false-positive rates resulting from miRNA target prediction. To distinguish positive disease-related miRNAs from negative ones, Xu *et al*.^14^ and Jing *et al*.^15^ extracted different features and presented the support vector machine classification approach. Jiang *et al*.^16^ developed a computational framework based on naive Bayes to mine underlying relationships from genomic data. However, negative samples of disease-related miRNAs are difficult even impossible to obtain^17^. These machine-learning-based approaches use unlabeled miRNA-disease associations as negative samples; inevitably, their accuracy of prediction is markedly influenced. Without using negative samples, Chen *et al*.^18^ proposed a semi-supervised approach, named Regularized Least Squares for miRNA-Disease Association (RLSMDA), which predicted miRNA-disease association on the framework of regularized least squares.

As summarized in the paper reviewed by Zhou *et al*.^19^, network similarity-based methods can be divided into two cases: local network similarity-based methods and global network methods. Xuan *et al*.^20^ proposed a locally network-based approach named HMDP based on weighted k of most similar neighbors to detect promising miRNA candidates for investigation of diseases. Computation strategies of miRNA functional similarity were improved in their study by integrating information on disease phenotype similarity, miRNA family, and clusters. Chen *et al*.^21^ first applied a global network-based method and advanced a method based on Random Walk with Restart (RWRMDA) for prediction of miRNA-disease associations. Li *et al*.^22^ proposed a computational model named MCMDA,which predicts the associations score of each miRNA-disease pair based on matrix completion.Chen *et al*.^23^ developed a novel method named miREFRWR based on the framework of random walk with restart to predict potential interactions between disease and miRNA-environmental factor. Chen *et al*.^24^ advanced the miREFScan, which is a novel prediction approach based on semi-supervised classifier, to predict underlying disease-related associations between miRNAs and environmental factors(EFs). miREFScan is the first computational approach to predict correlation among miRNAs, EFs, and diseases simultaneously. These approaches performed well in cross validation. However, they cannot be used for diseases without known related miRNAs.

Chen *et al*.^25^ proposed Network-Consistency-Based Interface (Net-CBI), another global-based approach, to identify underlying miRNA-disease associations. Net-CBI can isolate disease prediction, but its predictive performance is significantly poorer than that of RWRMDA. By combining multiple data sources, Liu *et al*.^26^ constructed a heterogeneous network to predict disease-related miRNAs. Chen *et al*.^27^ proposed a method called Restricted Boltzmann machine for multiple types of miRNA-disease association prediction to predict multi-type miRNA-disease relationships.Chen *et al*.^28^ proposed a new approach named WBSMDA, which predicts miRNA-disease interactions based on the model of within and between score. By integrating experimentally validated miRNA-disease associations and various similarity information based on miRNA and disease into a heterogeneous graph, Chen *et al*.^29^ proposed HGIMDA based on the framework of heterogeneous graph inference to reveal potential associations between miRNA and disease. You *et al*.^30^ proposed a path-based prediction model, named PBMDA, to infer underlying miRNA-disease associations. By integrating various reliable biological datasets, PBMDA constructs a heterogeneous graph and applies depth-first search algorithm in the integrated heterogeneous network. Chen *et al*.^31^ developed a new computational approach named SDMMDA based on super-disease and super miRNA to predict underlying miRNA-disease interactions. Chen *et al*.^32^ proposed a new computational model of ranking-based KNN named RKNNMDA to discover potential relationship between miRNAs and diseases.

As a whole, limitations of previous approaches can be summarized as follows. First, several methods strongly rely on uncertain data, such as miRNA-target associations. Second, several machine-learning-based approaches require negative samples, which are difficult to obtain. Third, several approaches work ineffectively on isolated diseases. Finally, certain approaches, such as Net-CBI, perform poorly in predicting isolated diseases.

To overcome the above deficiency, we developed Improved Low-Rank Matrix Recovery (ILRMR) for prediction of miRNA-disease association. The algorithm of matrix recovery is widely used in recommender systems, shows good prediction performance^33, 34^, and is successfully applied in other fields, such as movie, commodity, and social tags^35–37^. Our method combines multiple biological data and is based on the hypothesis that similar miRNAs interact with similar diseases. Cross validation and case studies showed that ILRMR performs better compared with other methods.

The main contributions of this study are as follows:

1. ILRMR is a semi-supervised learning approach that overcomes obstacles in obtaining negative samples in practical problems.
2. Various biological data are integrated into matrix recovery to precisely capture new underlying associations; these data constitute similarities between miRNA-miRNA and disease-disease, miRNA family information, and known correlations between miRNA and disease.
3. This study improves computational strategies on miRNA similarities and disease similarities.
4. ILRMR is a global approach that can predict all disease simultaneously and have the ability to new disease without known link to miRNAs.

## Results

### Performance evaluation of ILRMR

In this section, we adopt two approaches to evaluate predictive performance of ILRMR: (1) Leave-one out cross validation (LOOCV) was implemented for ILRMR by using a benchmark based on known and experimentally verified miRNA-disease associations. In LOOCV of ILRMR, each known miRNA-disease interaction was excluded as test sample, and remaining interactions were used as training samples to recover predictive matrix. (2) To further prove robustness of ILRMR, we masked portions of interactions according to mask ratios in experiments and evaluated recovery and prediction ability of ILRMR. In comparison between methods, area under the receiver operating curve (ROC) (AUC) value was calculated as performance criterion of evaluation. An AUC value that closely approximates 1 indicates a significantly improved performance. ROC curve^38^ plots the true positive rate or sensitivity versus false positive rate or 1-specificity at different thresholds. Sensitivity refers to ratio of correctly predicted interactions to total experiment-verified miRNA-disease interactions. Specificity refers to proportion of interactions below the given threshold. However, considering a large number of unlabeled associations in the dataset, using only AUC to assess the predictive performance of the model was not insufficient. The area under precision-recall curve (AUPR) is as much as possible to reduce the affect on predictive performance caused by false positive data. Thus, using AUC and AUPR value to evaluate the performance can be more reasonable.

**Figure 1:**
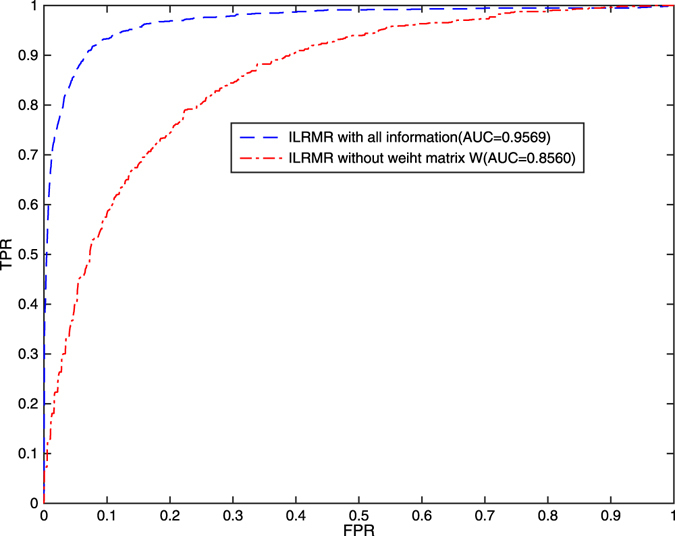
Performance evaluation of ILRMR in two situations based on LOOCV. (1) ILRMR with all information (ILRMR); (2) ILRMR without weight matrix *W*.

Based on multi-information, including miRNA-functional similarity, miRNA cosine-based similarity, miRNA family information, and disease semantic similarity, ILRMR integrates weight matrix W to recover association matrix. We evaluate predictive performance of ILRMR while considering the following aspects: (1) ILRMR with all information and (2) ILRMR without weight matrix W. Figure 1 plots the ROC curve of the two situations mentioned above.

ILRMR exhibited a commendable performance, and AUC values in the two situations reached 0.8560 and 0.9569, respectively. AUC value increased by 10.09% compared with ILRMR without weight matrix W. Evidently, weight matrix W based on miRNA (disease) similarity benefits improvement of predictive performance of ILRMR.

To further evaluate predictive performance of ILRMR, we assume that known miRNA-disease association matrix is complete and mask part of associations according to its mask ratio. The masked association matrix *X* = [*x*_1_, *x*_2_, …, *x*_n_], in which only part of associations are kept, was recovered by ILRMR. We varied mask ratios from 0.1 to 0.6 for each sample and with an interval of 0.05. We implemented experiments for 20 times and calculate average performance. Table 1 summarizes performance of ILRMR under different mask ratios in terms of AUC. Results demonstrate that robust ILRMR performs reliably and efficiently mines potential miRNA-disease associations when the numbers of known associations decrease. AUC values markedly declined when mask ratio increased from 0.1 to 0.6. However, the value remains considerable.

**Table 1:** Comparison of prediction performance of ILRMR under different mask ratios.

**Mask Ratio**	0.10	0.15	0.20	0.25	0.30	0.35	0.40	0.45	0.50	0.55	0.60
**AUC**	0.9432	0.9371	0.9280	0.9188	0.9067	0.8846	0.8769	0.8536	0.8301	0.8276	0.8014

### Comparison with other methods

To our knowledge, advanced computational approaches in miRNA-disease association prediction include RWRMDA^5^, Net-CBI^25^, HDMP^20^, RLSMDA^18^ and the global network method presented by Shi *et al*.^12^. However, RWRMDA and HDMP are local approaches that cannot work on diseases without known related miRNAs. Therefore, these approaches cannot be used for comparisons in this work. Considering that the method presented by Shi *et al*. predicted miRNA-disease association by integrating miRNA-targets association, disease gene associations, and protein interaction, the datasets totally different from the ones used in ILRMR. Moreover, known miRNA-disease associations were not used with their corresponding methods. Hence, this method cannot be reasonably and fairly compared with ILRMR. ILRMR, Net-CBI and RLSMDA all use similar data sets and can predict novel miRNA-disease associations for isolated diseases. In this view, we consider their performances for comparison.

**Figure 2:**
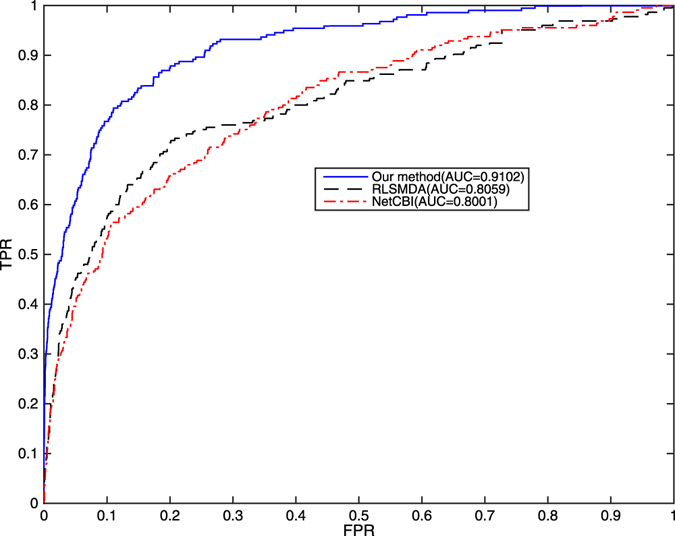
Comparison of methods between ILRMR, Net-CBI, and RLSMDA in terms of ROC curve and AUC on LOOCV. Without miRNA family and similarity of known miRNA-disease association network under consideration, ILRMR outperformed Net-CBI and RLSMDA in LOOCV.

**Figure 3:**
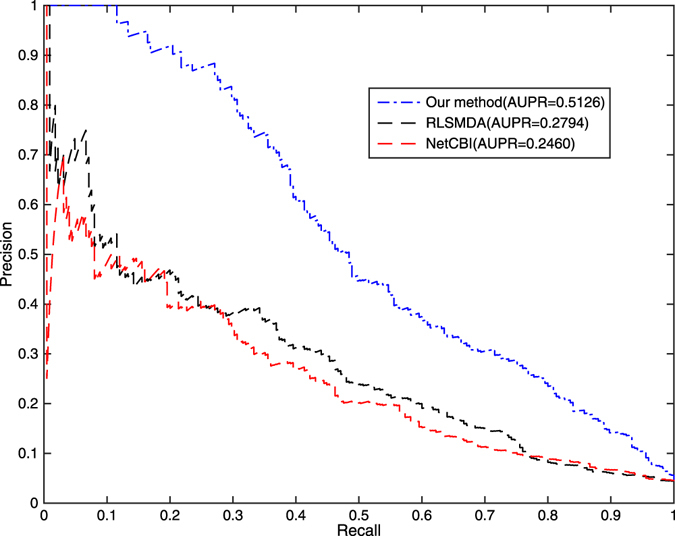
PR curve and AUPR values of ILRMR, RLSMDA and NetCBI.

We implement LOOCV on the benchmark to assess predictive performance of ILRMR, Net-CBI, and RLSMDA. Optimal parameters of Net-CBI and RLSMDA were set as described in corresponding literature. Considering the miRNA family information and the similarity of known miRNA-disease association have not been used in the method of NetCBI and RLSMDA, the three approaches were implemented only using miRNA functional similarities and disease semantic similarities in the comparisons of predicting. Figure 2 shows ROC curve and AUC value of the three methods. Without miRNA family information and cosine-based similarity of miRNA and disease under consideration, ILRMR achieved a reliable AUC of 0.9102. Net-CBI and RLSMDA achieved AUC values of 0.8001 and 0.8059, respectively. Figure 3 shows precision-recall curve and AUPR values of ILRMR, RLSMDA and NetCBI. Evidently, ILRMR outperformed Net-CBI and RLSMDA in LOOCV.

To further prove the strength of algorithms and avoid data dependence, we also implement LOOCV on predictive dataset. An AUC value of 0.9675 was obtained from ILRMR without considering miRNA family information and similarity of known miRNA-disease association network presented. Net-CBI and RLSMDA obtained AUC values of 0.9511 and 0.9560, respectively.

### Case studies

To further evaluate the ability of ILRMR to predict underlying disease-related miRNA candidates, we analyze case studies on lung and breast cancers. All known miRNA-disease associations in predictive dataset were used as training set to predict potential disease-related miRNA candidates based on the ILRMR model. Predictive results were verified based on the latest version of HMDD^7^. We also check results on updated miRNA-disease relevant database, miR2Disease^8^, and dbDEMC^9^. Table 2 and Supplementary Information S3 respectively list the top 50 lung cancer-related and breast cancer-related miRNAs predicted by ILRMR and confirmations of these associations.

**Table 2:** The top 50 lung cancer-related miRNAs predicted by ILRMR and the confirmation of these associations.

rank	miRNA name	evidences	rank	miRNA name	evidences
1	hsa-mir-31	HMDD, dbDEMC, miR2disease	26	hsa-mir-204	miR2disease
2	hsa-mir-542	HMDD	27	hsa-mir-25	HMDD, dbDEMC
3	hsa-mir-222	HMDD, dbDEMC, miR2disease	28	hsa-mir-133a	HMDD, dbDEMC
4	hsa-mir-494	HMDD, dbDEMC	29	hsa-mir-429	dbDEMC, miR2disease
5	hsa-mir-103	HMDD, dbDEMC	30	hsa-mir-339	dbDEMC, miR2disease
6	hsa-mir-7	HMDD, miR2disease	31	hsa-mir-127	HMDD,dbDEMC
7	hsa-mir-10b	HMDD, dbDEMC	32	hsa-mir-215	dbDEMC
8	hsa-mir-93	HMDD, dbDEMC, miR2disease	33	hsa-mir-451	dbDEMC, miR2disease
9	hsa-mir-221	HMDD,dbDEMC	34	hsa-mir-302c	dbDEMC
10	hsa-mir-141	dbDEMC, miR2disease	35	hsa-mir-151	Unconfirmed
11	hsa-mir-99a	dbDEMC, miR2disease	36	hsa-mir-373	dbDEMC
12	hsa-mir-296	dbDEMC	37	hsa-mir-130a	dbDEMC, miR2disease
13	hsa-mir-23a	HMDD, dbDEMC	38	hsa-mir-200c	HMDD, dbDEMC, miR2disease
14	hsa-mir-16	dbDEMC, miR2disease	39	hsa-mir-15b	dbDEMC
15	hsa-mir-98	HMDD, dbDEMC, miR2disease	40	hsa-mir-18b	HMDD
16	hsa-mir-488	dbDEMC	41	hsa-mir-135b	HMDD, dbDEMC
17	hsa-mir-302d	dbDEMC	42	hsa-mir-372	Unconfirmed
18	hsa-mir-200b	HMDD,dbDEMC, miR2disease	43	hsa-mir-27a	HMDD, dbDEMC
19	hsa-mir-200a	HMDD, dbDEMC, miR2disease	44	hsa-mir-423	miR2disease
20	hsa-mir-185	HMDD, dbDEMC	45	hsa-mir-22	HMDD, dbDEMC, miR2disease
21	hsa-mir-320	dbDEMC	46	hsa-mir-107	HMDD, dbDEMC
22	hsa-mir-377	miR2disease	47	hsa-mir-20b	dbDEMC
23	hsa-mir-195	dbDEMC, miR2disease	48	hsa-mir-376b	Unconfirmed
24	hsa-mir-181b	HMDD, dbDEMC, miR2disease	49	hsa-mir-629	HMDD
25	hsa-mir-135a	HMDD, dbDEMC	50	hsa-mir-486	HMDD, dbDEMC

Forty-seven of the top 50 potential lung cancer miRNAs candidates have been confirmed based on the update HMDD, dbDEMC and mir2disease.

Lung cancer is one of the most common malignant tumors with the highest morbidity and mortality and heavily threatens people’s health and life. In the predictive dataset, we discover 72 miRNAs related to lung cancer. Underlying lung cancer-related miRNA candidates were predicted by ILRMR based on 72 known associations. Table 2 provides the top 50 lung cancer-related miRNAs predicted by ILRMR. One typical example is hsa-miR-31, which ranked first in predictive results. Recent studies^39^ demonstrated close connection of miRNAs to clinicopathological parameters in clinical stages of lung cancer. Hsa-mir-31 expression significantly increases in lung cancer patients with poor survival^40^. Among the top 50 prediction list, 47 miRNAs were verified by HMDD, dbDEMC, and miR2Disease; and only hsa-mir-151, hsa-mir-372, and hsa-mir-376b were not confirmed. However, Leidinger P. *et al*.^41^ demonstrated that hsa-mir-151 is upregulated in non-small cell lung carcinoma compared with non-tumorous tissues. As described in literature^42^, T. Nijjar *et al*. identified that low expression level of hsa-mir-372 can be associated with recurrence case groups of stage I of non-small cell lung cancer. Evidence supported by literature further confirms reliability of ILRMR in predicting new underlying disease-related miRNA candidates.

Breast cancer is a malignant tumor that occurs in glandular epithelium of breasts and is regarded as the first major harm to women’s health^43^. In the predictive dataset, 78 miRNAs are related to breast cancer. As shown in Supplementary Table 1, 48 of the top 50 breast cancer-related miRNA candidates predicted by ILRMR were confirmed by the three databases mentioned above. For example, hsa-mir-340^44^, which ranked first in the predictive list, inhibits migration and development of breast cancer cell by targeting oncoprotein c-Met. hsa-mir-301a and hsa-mir-301b^45^ ranked third and ninth, respectively; they are pivotal oncogenes in human breast cancer and promote nodal or distant relapses through multiple pathways. hsa-mir-7^46, 47^ family is regarded as tumor suppressor to migration of breast cancer. In our experiment, hsa-mir-7i, hsa-mir-7b, and hsa-mir-7g are ranked first in the top 10 list.

### Applicability of ILRMR to predict diseases without any known associated miRNAs

To further verify the ability of ILRMR to predict diseases without any known associated miRNAs, we removed known verified miRNAs-disease associations on predictive diseases mentioned in the predictive dataset. This procedure ensured that prediction only considered similar information and known miRNA-disease association of other diseases. We deployed case studies for lung cancer and breast cancer, and predictive results are listed in Supplementary Table 2 and Table 3, respectively. For lung cancer, we removed 72 known miRNA-lung-cancer-related associations to predict underlying associations by ILRMR. Among the top 50 potential lung cancer miRNA candidates, 48 were based on recently updated HMDD, dbDEMC, and miR2Disease. For breast cancer, 78 known associations related to breast cancer were removed, and 47 of the top 50 predicted miRNAs were verified. The top 30 predictions for lung cancer and breast cancer were all confirmed. Therefore, ILRMR exhibits excellent performance in predicting diseases without known associated miRNAs. Topical subheadings are allowed.

**Table 3:** miRNA-disease associations.

miRNA	disease			
	*d*_1_	*d*_2_	…	*d*_*n*_
*m*_1_	0	1	…	0
*m*_2_	1	0	…	0
…	0	0	…	1
*m*_*m*_	1	0	…	0

### Application of ILRMR to predict novel human miRNAs-disease associations

The reliable performance of our algorithm had been thoroughly verified on cross validation and case studies as discussed above. Here, we further demonstrated the application of ILRMR to globally predict new potential miRNA-disease associations. All the known miRNA-disease associations in the predictive dataset were used for prediction. We ranked the unknown associations according to the scores recovered by ILRMR, and manually verified the top 50 associations through three updated HMDD, miR2diseases and dbDEMC. The predictive results and confirmations of these associations are listed in Supplementary Table 4.

## Discussion

Revelation of potential relationship between diseases and miRNAs is a critical problem not only in uncovering molecular mechanisms of various diseases but also in providing underlying biomarkers for disease diagnosis, treatment, and drug design. In this paper, we develop ILRMR for miRNA-disease association prediction. Compared with other state-of-the-art computational methods, ILRMR is a global method that can simultaneously prioritize potential associations of all diseases and does not require negative samples. ILRMR can also identify promising miRNAs for investigating diseases without any known related miRNA. By integrating miRNA-miRNA similarity information, disease-disease similarity information, and miRNA family information to matrix recovery, ILRMR performs better compared with other methods in cross validation and case studies.

Reliable performance of ILRMR can be majorly attributed to combination of the following algorithm factors. (1) This algorithm integrates various biological information, specifically on similarities of miRNA and disease, to matrix recovery, thereby significantly improving prediction performance. (2) The algorithm takes full advantages of unlabeled data in the miRNA-disease association matrix. (3) ILRMR solved by augmented Lagrange multipliers (ALMs) shows good convergence to obtain optimal solutions^48^.

ILRMR can be a valuable computational tool for predicting miRNA-disease associations. This approach can be further applied to reveal other biological associations, such as IncRNA-disease, gene-disease, and drug-target associations. However, the proposed approach also presents several limitations. First, a more reasonable construction of weight matrix based on miRNA similarity and disease similarity will further improve prediction capabilities. Second, further work can be conducted to extend similarity measures as a regression and to make the model more efficient and general.

Wang *et al*. ^49–51^ discussed a cancer hallmark network framework and cancer systems biology in the genome sequencing era. It is very interesting and so instructive for our in-depth analysis and understanding of the pathogenesis of cancer. At present, we predicted only whether there is an association between miRNAs and diseases. The specific regulation mechanism has not yet been studied. Whether the miRNAs regulate more cancer hallmark genes deserves a closer look. From this perspective, more research work we may able to carry out in the future work.

## Methods

### Data Preparation

Data on miRNA-disease associations used in this paper were obtained from HMDD constructed by Li *et al*. ^7^. Two versions (September-2009 Version and V2.0 Version) of HMDD associations were used in our study. The first version was used as predictive dataset to predict new miRNA-disease associations. The latest version was used to confirm prediction results. Two other online databases, miR2Disease and dbDEMC, which were constructed by Jiang *et al*.^8^ and Yang *et al*.^9^, were also used for confirmation of predicted results. To further demonstrate generalization abilities of our methods for certain situations, that is, extremely limited known and experimentally identified miRNA-disease interactions, miRNA-disease association data from ref. 11 were also used as benchmark datasets in the paper. miRNA functional similarity scores were downloaded from http://www.cuilab.cn, which is a reliable website that provides biological data to facilitate research for biologists and medical scientists. Disease semantic similarities were calculated similarly as those in other studies^52^, whereas similarity score can be obtained from supplementary material in ref. 18.

### Problem Description

We considered *m* miRNAs and *n* diseases and supposed that original matrix *A*_*m*×*n*_ represents adjacency matrix of miRNA-disease association, where *A*_*ij*_ = 1 is the *i* ^*th*^ miRNA that interacts with the *j* ^*th*^ disease; otherwise, *A*_*ij*_ = 0. As shown in Table 3, a value of 1 represents corresponding miRNA-disease association verified though biological experiments and exists in databases, including mir2Disease, HMDD, and dbDEMC. A value of 0 represents a missing value (unknown association that will be predicted). The masked association matrix *X*_*m*×*n*_ is obtained from the original association matrix *A*_*m*×*n*_, and masked part of interaction according the mask ratio demanded in the cross validation. The work we need to do is to estimate the missing value of the matrix based on the existing association and relevant information.

### Model of LRMR for predicting miRNA-disease association

Low-rank matrix recovery (LRMR) is a highly effective algorithm for predicting missing values. This algorithm uses different mathematical or machine learning methods to decompose potential characteristics from an original matrix to explain and to predict missing values. Limited validated numbers are available for known miRNA-disease associations through biological experiments, whereas negative samples are difficult or impossible to obtain. Matrix *A*_*m*×*n*_ of miRNA-disease association is sparse and imbalanced. Furthermore, a certain degree of potential similarity exists among column (row) vectors in association matrix. Given the characteristics of association matrix mentioned above, we considered recovery of matrix by using robust principal component analysis (rPCA), which is one of the powerful models used in ILRMR.

We predicted unknown miRNA-disease associations based on the robust PCA model by using (1), which minimizes errors between known association matrix *X* and resuming matrix *R*_*mir*_*dd*_:

1 $$\mathop{{\rm{\min }}}\limits_{{R}_{mir\_dd},E}{\Vert {R}_{mir\_dd}\Vert }_{\ast }+\lambda {\Vert E\Vert }_{1},\,\,subject\,to\,\,X={R}_{mir\_dd}+E$$

where ${\Vert {R}_{mir\_dd}\Vert }^{\ast }$ denotes the nuclear norm^53^ of the resuming miRNA-disease association matrix *R*_*mir*_*dd*_, ${\Vert E\Vert }_{1}$ reprents the ${\ell }_{1}-norm$ of the discrepancy matrix *E*, weight parameter *λ* denotes weight sparse error term in the cost function, and 0 ≤ *λ* ≤ 1. Optimization model can be solved using the exact ALM method from a previous study^48^.

### Calculating miRNA-based similarity

To improve the accuracy of association matrix recovery and the prediction effects, we combined the weight matrix *W* with the robust PCA model, which includes miRNA-miRNA similarity and disease-disease similarity. By Comparing with a similarity measure method in a previous study^52, 54^, we calculate miRNA similarity by integrating multi-information, including miRNA functional similarity, cosine-based similarity, and miRNA family information. Considering each miRNA as a vector of the frequency of the interaction with the diseases, we then computed the cosine value of the angle formed by two miRNA vectors^55^. Assuming that *Sim*_*mir*_*cos*_ represents the miRNA similarity matrix, we calculate cosine-based similarity by (2)

2 $$Si{m}_{mir\_cos}(i,j)=\frac{{x}_{i}{x}_{j}^{T}}{\Vert {x}_{i}\Vert \parallel {x}_{j}\parallel }$$

Then, we integrate the miRNA functional similarity (matrix *Sim*_*mir*_*fun*_), information on miRNA families (matrix *Sim*_*FAM*_) and cosine-base similarity(matrix *Sim*_*mir*_*cos*_) into Eq. (3) to construct the final miRNA-miRNA similarity:

3 $$Si{m}_{mir}(i,j)=Si{m}_{mir\_fun}(i,j)\times \mathrm{(1}+Si{m}_{mir\_cos}(i,j))\times \mathrm{(1}+FAM(i,j))$$

miRNA functional similarity score calculation was based on the method proposed by Wang^52^. miRNA family information was obtained from the miRBase database^56^. When miRNAs *i* and *j* belong to the same family, value of *FAM*(*i*, *j*) is 1, otherwise the value is 0. *Sim*_*mir*_(*i*, *j*) denotes the final similarity score between miRNA *i* and *j*. When *i* ^*th*^ and *j* ^*th*^ miRNAs are more similar and belong to the same family, *Sim*_*mir*_(*i*, *j*) is higher.

### Calculating disease-based similarity

Similar to the calculations of miRNA cosine-based similarity, disease cosine-based similarity was computed. We assume that *X* = [*x*_1_, *x*_2_, …, *x*_*n*_] represents the miRNA-disease association matrix. *Sim*_*dd_cos*_ represents similarity matrix between diseases according to known correlations in the miRNA-disease associaton network. We calculate the matrix by (4) based on the vector cosine-based similarity measure method:

4 $$Si{m}_{dd\_cos}(i,j)=\frac{{X}_{i}{X}_{j}^{T}}{\Vert {X}_{i}\Vert \parallel {x}_{j}\parallel }$$

where *X*_*i*_ denotes *i* ^*th*^ row of *X*.

Then, we integrate the disease semantic similarity and cosine-based similarity into Eq. (5) to construct final disease-disease similarity

5 $$Si{m}_{dd}(i,j)=Si{m}_{dd\_phe}(i,j)\times \mathrm{(1}+Si{m}_{dd\_cos}(i,j))$$

where *Sim*_*dd_phe*_(*i*, *j*) corresponds to the semantic similarity score of diseases *i* and *j*. From the Medical Subject Heading database (a strict system for disease description and classification), diseases were described in a DAG. Disease semantic similarity can be calculated based on the assumption that two diseases sharing more parts of DAGs are more similar^18^. *Sim*_*dd*_(*i*, *j*) represents the final similarity between diseases *i* and *j*. When two diseases are more similar, score is higher.

### Calculating weight matrix *W* based on miRNA(disease) similarity and prediction of novel association

**Figure 4:**
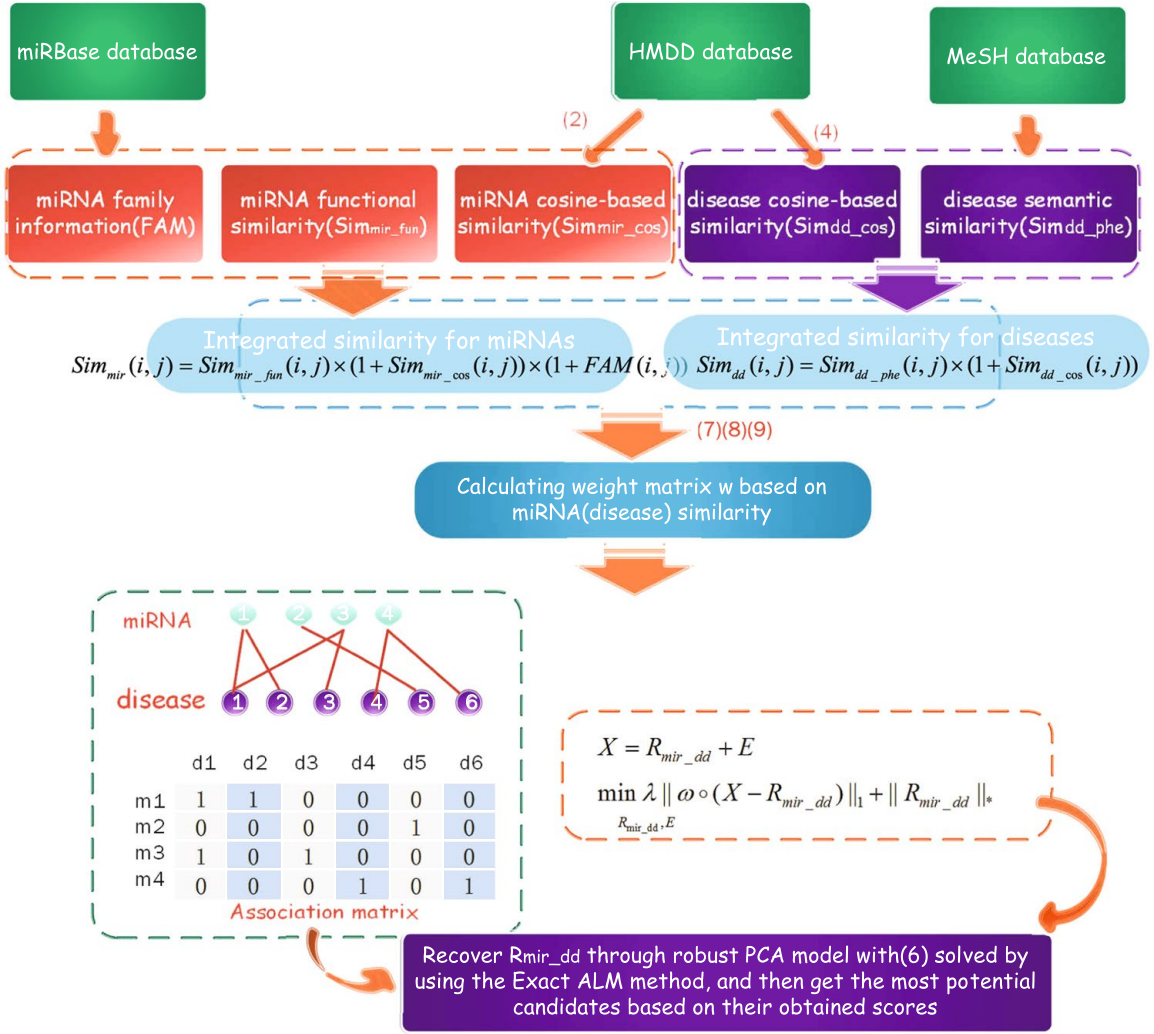
The overall flowchart of ILRMR.

To further improve the prediction accuracy, we integrate weight matrix *W* based on the miRNA and disease similarity mentioned above to matrix recovery algorithm. Thus, we obtain the following prediction formula (6):

6 $$X={R}_{mir\_dd}+E\mathop{\min }\limits_{{R}_{mir\_dd},E}\lambda {\Vert W\circ (X-{R}_{mir\_dd})\Vert }_{1}+\Vert {R}_{mir\_dd}\Vert \ast $$

where *W* denotes weight matrix based on miRNA (disease) similarity. Figure 4 shows the overall flowchart of ILRMR method. The algorithm is summarized as follow.

In our method, association matrix *X*_*m*×*n*_ is decomposed into sum of low rank matrix *R*_*mir_dd*_ and sparse noise matrix *E*, and low rank matrix *R*_*mir_dd*_ is then recovered by solving the nuclear norm optimization problem. ${\ell }_{1}-norm$ is used to suppress noise. We used Hadamard product between weight matrix *W* and discrepancy matrix to improve accuracy of the recovery. Considering that matrix *X*_*m*×*n*_ measures *m*×*n* and Hadamard product^57^ is a class of matrix operation, in which operation of two matrices matrix must be of the same order, we calculate W by (7) based on calculation of similarity mentioned above with appropriate transformation:

7 $${W}_{(i,j)}=\frac{{W}_{mir}(i,j)+{W}_{dd}(i,j)}{2}$$

#### **Algorithm 1**

: ILRMR for miRNA-disease interaction prediction.

Weight matrix *W* consists of two parts, namely, *W*_*mir*_(*i*, *j*) and *W*_*dd*_(*i*, *j*), which weights based on miRNA-miRNA similarity and disease-disease similarity, respectively. *W*_*mir*_(*i*, *j*) denotes the miRNA-based similarity weight value, and is calculated by (8)

8 $${W}_{mir}(i,j)=\frac{Si{m}_{mi{r}_{i}}\times {X}_{j}}{{X}_{j}}$$

where $Si{m}_{mi{r}_{i}}$ corresponds to *i* ^*th*^ row of matrix *Sim*_*mir*_ and vector comprising the similarities between miRNA *i* and all other miRNAs. *X*_*j*_ denotes the *j* ^*th*^ column of association matrix *X*_*m*×*n*_ and the vector consisting of the interactions between disease *j* and all miRNAs. $|{X}_{j}|$ represents the length of vector *X*_*j*_ (the norm of vector *X*_*j*_). Evidently, higher value of *W*_*mir*_(*i*, *j*), indicates higher possibility that miRNA *i* is associated with disease *j*.

Similarly, *W*_*dd*_(*i*, *j*) denotes disease-based similarity weight value, and is calculated by (9)

9 $${W}_{dd}(i,j)=\frac{{X}_{i}\times Si{m}_{d{d}_{j}}}{|{X}_{i}|}$$

where $Si{m}_{d{d}_{j}}$ corresponds to *j* ^*th*^ column of matrix *Sim*_*dd*_ and the vector consisting of similarities between disease *j* and all other diseases. *X*_*i*_ corresponds to the *i* ^*th*^ row of matrix *X*_*m*×*n*_ and the vector consisting of interactions between miRNA *i* and all diseases. Notebly, higher value of *W*_*dd*_(*i*, *j*), indicates a higher probability that miRNA *i* is associated with disease *j*.

## Electronic supplementary material

Supplementary information
