# Supplementary material for: Improved low-rank matrix recovery method for predicting miRNA-disease association
Source: Sci Rep. 2017 Jul 20;7:6007. doi: 10.1038/s41598-017-06201-3 (PMC5519594; doi:10.1038/s41598-017-06201-3)
Supplement: Supplementary file 1 — Supplementary information [file 41598_2017_6201_MOESM1_ESM.pdf]

Improved low-rank matrix recovery method for predicting miRNA-disease association

Li Peng[1,2], Manman Peng[1,*], Bo Liao[1], Guohua Huang[3], Wei Liang[2], and Keqin Li[4]
[1]College of Information Science and Engineering, Hunan University, Changsha, Hunan, 410082, China
[2]College of Computer Science and Engineering, Hunan University of Science and Technology, Xiangtan, Hunan,411201, China
[3]College of Information Engineering, Shaoyang University, Shaoyang, Hunan,422000,China
[4]Department of Computer Science, State University of New York, New Paltz, New York 12561, USA
*pengmanman@hnu.edu.cn

Supplementary Information

Supplementary Table 1. The top 50 breast cancer-related miRNAs candidates predicted by ILRMR and the confirmation of these associations. Forty-eight of the top 50 potential breast cancer miRNAs candidates have been confirmed based on the update HMDD, dbDEMC and mir2disease.

rank	miRNA name	evidences	rank	miRNA name	evidences
**1**	hsa-mir-340	HMDD,dbDEMC	26	hsa-mir-193b	HMDD, dbDEMC, miR2disease
**2**	hsa-let-7i	HMDD,dbDEMC,miR2disease	27	hsa-mir-520h	HMDD
**3**	hsa-mir-301a	HMDD	28	hsa-mir-203	HMDD,mir2disease,dbDEMC
**4**	hsa-let-7b	HMDD, dbDEMC	29	hsa-mir-96	HMDD,dbDEMC, miR2disease
**5**	hsa-mir-328	HMDD,dbDEMC, miR2disease	30	hsa-mir-191	HMDD, dbDEMC, miR2disease
**6**	hsa-mir-99a	dbDEMC	31	hsa-mir-338	HMDD,dbDEMC
**7**	hsa-mir-101	HMDD,dbDEMC, miR2disease	32	hsa-let-7c	HMDD,dbDEMC
**8**	hsa-mir-7g	HMDD,dbDEMC	33	has-mir-27a	HMDD, dbDEMC, miR2Disease
**9**	hsa-mir-301b	HMDD	34	hsa-mir-31	HMDD, dbDEMC, miR2disease
**10**	hsa-mir-28	dbDEMC	35	hsa-mir-16	HMDD, dbDEMC
**11**	hsa-mir-29c	HMDD,dbDEMC,miR2disease	36	hsa-mir-27b	HMDD, dbDEMC
**12**	hsa-mir-92a	HMDD	37	hsa-mir-98	dbDEMC, miR2disease
**13**	hsa-mir-24	HMDD,dbDEMC	38	hsa-mir-212	dbDEMC
**14**	hsa-mir-183	HMDD,dbDEMC	39	has-mir-126	HMDD,dbDEMC,miR2Disease,
**15**	hsa-let-140	HMDD,dbDEMC	40	hsa-mir-542	Unconfirmed
**16**	hsa-mir-152	HMDD,dbDEMC, miR2disease	41	hsa-mir-372	dbDEMC
**17**	hsa-mir-612	dbDEMC	42	hsa-mir-181a	HMDD,dbDEMC, miR2Disease
**18**	hsa-mir-181c	dbDEMC	43	has-mir-373	HMDD,dbDEMC, miR2Disease
**19**	hsa-mir-18b	HMDD,dbDEMC	44	hsa-mir-182	HMDD,dbDEMC, miR2Disease
**20**	hsa-mir-224	HMDD,dbDEMC,	45	hsa-mir-106a	dbDEMC
**21**	has-mir-100	dbDEMC	46	hsa-mir-137	HMDD,dbDEMC
**22**	hsa-mir-15b	dbDEMC	47	hsa-mir-514	Unconfirmed
**23**	hsa-mir-182	HMDD,dbDEMC,miR2Disease	48	hsa-mir-130a	dbDEMC
**24**	hsa-mir-128b	miR2Disease	49	has-mir-150	dbDEMC
**25**	has-mir-22	HMDD,dbDEMC,miR2Disease	50	hsa-mir-124	HMDD, dbDEMC, miR2disease

Supplementary Table 2. The top 50 lung cancer-related miRNAs predicted by ILRMR and the confirmation of these associations. Forty-eight of the top 50 potential lung cancer miRNAs candidates have been confirmed based on the update HMDD, dbDEMC and mir2disease.

rank	miRNA name	evidences	rank	miRNA name	evidences
1	hsa-let-7g	HMDD,dbDEMC,miR2disease	26	hsa-mir-302d	dbDEMC
2	hsa-mir-140	HMDD,dbDEMC,miR2disease	27	hsa-mir-145	HMDD, dbDEMC
3	hsa-mir-195	dbDEMC, miR2disease	28	hsa-mir-27a	HMDD, dbDEMC
4	hsa-mir-17	HMDD, miR2disease	29	hsa-mir-16	dbDEMC, miR2disease
5	hsa-mir-218	HMDD,dbDEMC,miR2disease	30	hsa-mir-124	HMDD, miR2disease
6	hsa-mir-34b	HMDD, dbDEMC	31	hsa-mir-21	HMDD,dbDEMC,miR2disease
7	hsa-mir-103	HMDD,dbDEMC	32	hsa-mir-29a	HMDD,dbDEMC,miR2disease
8	hsa-mir-429	dbDEMC, miR2disease	33	Has-mir-320	dbDEMC
9	hsa-mir-130a	dbDEMC, miR2disease	34	hsa-mir-203	HMDD,dbDEMC,miR2disease
10	hsa-mir-15b	dbDEMC	35	hsa-mir-130b	dbDEMC
11	hsa-mir-221	HMDD,dbDEMC	36	hsa-mir-302c	dbDEMC
12	hsa-mir-9	HMDD, miR2disease	37	hsa-mir-185	HMDD, dbDEMC
13	hsa-mir-22	HMDD, miR2disease	38	hsa-mir-155	HMDD,dbDEMC,miR2disease
14	hsa-mir-135b	HMDD, dbDEMC	39	hsa-mir-99a	dbDEMC, miR2disease
15	hsa-mir-92a	HMDD	40	hsa-mir-34a	HMDD, dbDEMC
16	hsa-mir-146a	HMDD,dbDEMC,miR2disease	41	hsa-mir-20b	dbDEMC
17	hsa-mir-7	HMDD,dbDEMC,miR2disease	42	hsa-mir-18b	HMDD
18	hsa-mir-24	HMDD, miR2disease	43	hsa-mir-18a	HMDD,dbDEMC,miR2disease
19	hsa-mir-10b	HMDD, dbDEMC	44	hsa-mir-373	dbDEMC
20	hsa-mir-25	HMDD, dbDEMC	45	hsa-mir-222	HMDD,dbDEMC
21	hsa-mir-27b	HMDD, miR2disease	46	hsa-mir-193	HMDD, dbDEMC
22	hsa-mir-451	dbDEMC, miR2disease	47	Has-mir-532	Unconfirmed
23	hsa-mir-423	miR2disease	48	hsa-mir-135a	HMDD, dbDEMC
24	hsa-mir-106a	HMDD, miR2disease	49	has-mir-107	HMDD, dbDEMC
25	hsa-let-7f	HMDD, , miR2disease	50	has-mir-210	HMDD,dbDEMC,miR2disease

Supplementary Table 3. The top 50 breast cancer-related miRNAs candidates predicted by ILRMR and the confirmation of these associations. Forty-seven of the top 50 potential breast cancer miRNAs candidates have been confirmed based on the update HMDD, dbDEMC and mir2disease.

rank	miRNA name	evidences	rank	miRNA name	evidences
**1**	hsa-mir-200a	HMDD, miR2disease	26	has-mir-608	HMDD, dbDEMC
**2**	hsa-mir-205	HMDD, miR2disease	27	hsa-mir-299	dbDEMC
**3**	hsa-let-7i	HMDD,dbDEMC,miR2disease	28	has-mir-367	HMDD
**4**	hsa-mir-99a	dbDEMC	29	hsa-mir-182	HMDD,dbDEMC,miR2Disease
**5**	hsa-mir-206	HMDD, miR2disease	30	hsa-mir-199a	HMDD,dbDEMC
**6**	hsa-mir-17	HMDD, miR2disease	31	hsa-let-7a	HMDD, miR2disease

**7**	hsa-let-7b	HMDD, dbDEMC	32	hsa-mir-98	dbDEMC, miR2disease
**8**	hsa-mir-29c	HMDD,dbDEMC, miR2disease	33	hsa-mir-152	HMDD,dbDEMC, miR2disease
**9**	hsa-mir-125a	HMDD, miR2disease	34	hsa-mir-18b	HMDD,dbDEMC
**10**	hsa-let-7f	HMDD, miR2disease	35	hsa-mir-132	HMDD,dbDEMC
**11**	hsa-let-7d	HMDD, miR2disease	36	hsa-mir-362	Unconfirmed
**12**	hsa-mir-200c	HMDD , miR2disease	37	hsa-mir-24	HMDD,dbDEMC
**13**	hsa-mir-340	HMDD,dbDEMC	38	hsa-mir-130b	dbDEMC
**14**	hsa-mir-101	HMDD,dbDEMC,miR2disease	39	hsa-mir-125b	HMDD, miR2disease
**15**	hsa-mir-181c	dbDEMC	40	hsa-mir-27b	HMDD, dbDEMC
**16**	hsa-mir-199b	HMDD,dbDEMC	41	hsa-mir-15b	dbDEMC
**17**	hsa-mir-301b	HMDD	42	hsa-mir-542	Unconfirmed
**18**	has-mir-27a	HMDD,dbDEMC,miR2Disease	43	hsa-mir-223	HMDD, dbDEMC
**19**	hsa-mir-144	dbDEMC	44	hsa-mir-200b	HMDD, miR2disease
**20**	hsa-mir-331	dbDEMC	45	hsa-mir-454	Unconfirmed
**21**	hsa-let-7g	HMDD,dbDEMC	46	hsa-mir-31	HMDD,dbDEMC,miR2disease
**22**	has-mir-126	HMDD,dbDEMC,miR2Disease	47	hsa-mir-520b	HMDD,dbDEMC
**23**	has-mir-148a	HMDD,dbDEMC,miR2Disease	48	hsa-mir-106a	HMDD,dbDEMC
**24**	hsa-mir-193b	HMDD,dbDEMC,miR2disease	49	hsa-mir-181a	HMDD,miR2Disease,dbDEMC
**25**	hsa-mir-429	HMDD, miR2disease	50	hsa-miR-150	HMDD, dbDEMC

Supplementary Table 4. The top 50 novel miRNA-diseases associations predicted by ILRMR and the confirmation of these associations.

rank	diseases name	miRNA name	evidences
**1**	Colonic Neoplasms	hsa-mir-183	dbDEMC,miR2Disease
**2**	Lung Neoplasms	hsa-mir-542	HMDD
**3**	Breast Neoplasms	hsa-let-7i	HMDD,dbDEMC,miR2disease
**4**	Stomach Neoplasms	hsa-mir-451	HMDD, miR2Disease
**5**	Lung Neoplasms	hsa-mir-222	HMDD,dbDEMC,miR2disease
**6**	Ovarian Neoplasms	hsa-mir-143	miR2Disease
**7**	Carcinoma, Hepatocellular	hsa-mir-155	HMDD,dbDEMC,miR2Disease
**8**	Breast Neoplasms	hsa-mir-301a	HMDD
**9**	Neoplasms	hsa-mir-222	HMDD, dbDEMC
**10**	Lung Neoplasms	hsa-mir-204	miR2disease
**11**	Colonic Neoplasms	hsa-mir-181b	dbDEMC,miR2Disease
**12**	Breast Neoplasms	hsa-mir-92a	HMDD
**13**	Melanoma	hsa-mir-141	HMDD, miR2disease
**14**	Lung Neoplasms	hsa-mir-7	HMDD,miR2disease
**15**	Breast Neoplasms	hsa-mir-301b	HMDD
**16**	Colorectal Neoplasms	hsa-mir-137	HMDD,miR2Disease
**17**	Breast Neoplasms	hsa-mir-612	dbDEMC
**18**	Ovarian Neoplasms	hsa-let-7d	HMDD, miR2disease
**19**	Pancreatic Neoplasms	hsa-mir-18a	HMDD,miR2Disease
**20**	Breast Neoplasms	hsa-let-7b	HMDD, dbDEMC

**21**	Carcinoma, Hepatocellular	hsa-mir-155	HMDD,dbDEMC,miR2Disease
**22**	Colonic Neoplasms	hsa-mir-214	dbDEMC
**23**	Adenocarcinoma	hsa-mir-363	unconfirmed
**24**	Neoplasms	hsa-mir-125b	HMDD,dbDEMC
**25**	Ovarian Neoplasms	hsa-mir-34a	HMDD
**26**	Breast Neoplasms	hsa-mir-128b	miR2Disease
**27**	Prostatic Neoplasms	hsa-mir-15b	HMDD,dbDEMC
**28**	Breast Neoplasms	hsa-mir-130a	dbDEMC
**29**	Carcinoma, Hepatocellular	hsa-mir-29b	HMDD,dbDEMC
**30**	Breast Neoplasms	hsa-mir-372	dbDEMC
**31**	Ovarian Neoplasms	hsa-mir-373	miR2Disease
**32**	Lung Neoplasms	hsa-mir-93	HMDD,dbDEMC,miR2disease
**33**	Neoplasms	hsa-mir-20b	HMDD
**34**	Breast Neoplasms	hsa-let-7c	HMDD,dbDEMC
**35**	Ovarian Neoplasms	hsa-let-7c	HMDD,miR2disease
**36**	Lymphoma	hsa-mir-9	dbDEMC
**37**	Melanoma	hsa-mir-125a	HMDD, mir2disease
**38**	Brain Neoplasms	hsa-mir-16	unconfirmed
**39**	Breast Neoplasms	hsa-mir-181a	HMDD,dbDEMC,miR2Disease
**40**	Lymphoma, B-Cell	hsa-mir-221	miR2Disease
**41**	Pancreatic Neoplasms	hsa-mir-145	HMDD,dbDEMC,miR2disease
**42**	Lung Neoplasms	hsa-mir-221	HMDD,dbDEMC
**43**	Melanoma	hsa-mir-155	HMDD
**44**	Neoplasms	hsa-mir-106a	HMDD, dbDEMC
**45**	Breast Neoplasms	hsa-mir-203	HMDD,dbDEMC,miR2disease
**46**	Carcinoma, Hepatocellular	hsa-mir-107	HMDD,dbDEMC,miR2Disease
**47**	Melanoma	hsa-mir-30c	unconfirmed
**48**	Breast Neoplasms	hsa-mir-27b	HMDD, dbDEMC
**49**	Colonic Neoplasms	hsa-mir-31	HMDD,dbDEMC,miR2Disease
**50**	Lung Neoplasms	hsa-mir-373	dbDEMC
